# Estimated Dietary Intake of Trace Metals from Swordfish Consumption: A Human Health Problem

**DOI:** 10.3390/toxics6020022

**Published:** 2018-04-03

**Authors:** Grazia Barone, Angela Dambrosio, Arianna Storelli, Rita Garofalo, Vito Pietro Busco, Maria Maddalena Storelli

**Affiliations:** 1 Biosciences, Biotechnlogies and Biopharmacological Department, University of Bari “Aldo Moro”—Strada Prov. le per Casamassima Km 3, 70010 Valenzano (BA), Italy; grazia.barone@uniba.it (G.B.); arianna.storelli@uniba.it (A.S.); rita.garofalo@uniba.it (R.G.); vitopietro.busco@uniba.it (V.P.B.); 2 Department of Emergency and Organ Transplant, University of Bari “Aldo Moro”—Strada Prov. le per Casamassima Km 3, 70010 Valenzano (BA), Italy; angela.dambrosio@uniba.it

**Keywords:** swordfish, heavy metals, health risk, PTWI, THQ

## Abstract

Trace element (Hg, Pb, Cd, Zn, Cu, Ni, and Cr) occurrence was determined in the muscle tissue of swordfish collected in the Mediterranean Sea to assess whether the intakes complied with the recommended levels for essential metals and permissible levels for toxic elements. Metals were analyzed by an atomic absorption spectrophotometer (Shimadzu AA 7000). The methodology of Target Hazard Quotient (THQ) was also evaluated. The ranking order of toxic metal concentration was Hg > Cd > Pb, while for essential elements the distribution pattern followed the sequence Zn > Cu > Ni > Cr. The Estimated Weekly Intakes (EWI) as well as THQ for Cd and Pb indicated that swordfish consumption did not pose a risk to human health, whereas the major concern was for Hg. Fish size-related changes in Hg concentrations resulted in high EWI and THQ values relative to larger fish consumption, implying a potential risk to human health. For consumer protection, catches of swordfish approximately above 44 kg should be avoided as these fish have a higher risk of containing toxic levels of Hg.

## 1. Introduction

Swordfish, *Xiphias gladius* L. 1758, the only living species belonging to the Xiphiidae family, is a pelagic fish of high commercial value. Characterized by specific biological traits, such as long life-span and fast growth, this apical predator has an intense metabolic activity leading to continuous supply of energy. As a result, the rate of predation and food intake is extremely high, features exacerbating contaminant bioaccumulation in its body [1,2,3]. One of the most worrying classes of chemical contaminants in terms of toxicological risk to humans is represented by heavy metals [4]. Elements such as mercury (Hg), lead (Pb), and cadmium (Cd) are extremely toxic even in trace amounts, whereas other metals such as zinc (Zn), copper (Cu), chromium (Cr), and nickel (Ni), defined as essential because play an important role in biological systems, can also produce toxic effects when present in excessive concentrations. Diet is the primary pathway for metal accumulation in the general population and the consumption of contaminated fish is a key food source of exposure in humans [5,6,7]. Therefore, there are many national and international regulations regarding seafood safety as well as several health protection organizations that provide guidelines on the intake of trace elements by consumers. This aspect becomes a primary matter of concern in the case of top predators because the metal accumulation throughout the food web tends to be intensified, constituting a risk to human health. A lot of studies have, in fact, reported high contamination levels in large predator fishes, leading to elevated exposure in consumers, especially for mercury [8,9]. The EFSA Panel on Contaminants in the Food Chain for certain vulnerable groups, such as women of childbearing age, pregnant and breastfeeding women, as well as young children, recommends restricted consumption of predator fish, including shark, tuna, and swordfish [10,11]. Consequently, it becomes imperative to clarify the status of chemical contamination of this important fishery product in order to ensure safety for consumers. This is especially relevant when the organisms come from marine areas overexposed to anthropogenic pressure. This is the case in the Mediterranean Sea, which, with its semi-enclosed marine area with reduced hydrodynamism and limited water exchange with the Atlantic Ocean, constitutes an ideal sink for contaminants. On the other hand, pollution and over-fishing are among the causes of the decline in the Mediterranean swordfish population, now classified as near-threatened in an overview of the conservation of Mediterranean fish [12]. In this picture, the main purposes of current study were (1) to estimate the levels of toxic (Hg, Pb, and Cd) and essential metals (Zn, Cu, Cr, and Ni) in the muscle tissue of Mediterranean swordfish; (2) to ascertain whether the concentrations were compliant with the maximum limits defined by legislation in different countries; (3) to evaluate the health risk posed by fish consumption, comparing the estimated intake with reference toxicological and nutritional values for each element; and (4) to examine how human exposure varies according to the consumption of different sizes of fish.

## 2. Materials and Methods

### 2.1. Sample Collection

Approximately 100 g of muscle tissue from the carcass anterior portion of 30 Mediterranean swordfish specimens (fishing location: FAO area 37, division 37.2.2) were obtained from an Italian fish trade company. The sampling methodology used was in accordance with the Commission Regulation (EU) N 644/2017 [13]. These muscle portions, packed in ice, were transported to an analytical chemical laboratory, homogenized, and kept in a deep freeze at −20 °C until analysis. Weight (kg) and lower jaw fork length (LJFL) measurement to the nearest cm and for each fish are illustrated in Table 1.

### 2.2. Chemical Analyses

The extractive analytical procedure and the instrumental conditions for determine metal concentrations have been described in detail elsewhere [14]. Briefly, aliquots (about 1.0–2.0 g) of the samples were digested to a transparent solution with a mixture of HNO_3_-HClO_4_ (8:3) for Cd, Pb, Zn, Cu, Cr, and Ni determination and with a mixture of H_2_SO_4_–HNO_3_ (1:1) for Hg. The completely digested samples were allowed to cool and diluted with deionized water according to the method recommended by the Official Italian Agencies [15]. The metals content was determined by an atomic absorption spectrophotometer (Shimadzu AA 7000, Milan, Italy Zn and Ni were analyzed by flame; Cd, Pb, Cr, and Cu by using a graphite furnace (high-density tube) (GFA-7000); and Hg by using a hydride vapor generator (HVG-1) after reduction by NaBH_4_ (Table S1).

### 2.3. Quality Control and Assurance

Reference tissue (Tort-2 Lobster Hepatopancreas, National Research Council of Canada, Ottawa, ON, Canada) was treated and analyzed in the same way as the samples. Results (Hg: 0.28 ± 0.03; Cd: 26.2 ± 2.4; Pb: 0.32 ± 0.18; Zn: 188 ± 12; Cu: 101 ± 13; Ni: 2.3 ± 0.23; Cr: 0.73 ± 0.16 μg g^−1^ dry weight) were in good agreement with the certified values (Hg: 0.27 ± 0.06; Cd: 26.7 ± 0.60; Pb: 0.35 ± 0.13; Zn: 180 ± 6; Cu: 106 ± 10; Ni: 2.5 ± 0.19; Cr: 0.77 ± 0.15 μg g^−1^ dry weight) and the standard deviation was low (*n* = 3), proving the good repeatability of the methods. The results for the standard reference material displayed recoveries of the elements ranging from 91 to 104% (*n* = 3). The limit of detection (LOD) (Hg: 5; Cd: 0.12; Pb: 10; Zn: 24; Cu: 26; Ni: 26; Cr: 5 ng g^−1^ wet weight) is defined as the concentration corresponding to three times the standard deviation of blanks and the standards of quantification (LOQ) are the following: Hg: 13; Cd: 0.40; Pb: 0.38; Zn: 87; Cu: 81; Ni: 79; Cr: 16 ng g^−1^ wet weight. Two blank samples were analyzed together with each sample batch. Metal concentrations in blanks were below the detection limits in all the analyses. Blanks and calibration standard solutions were analyzed in a similar way to the digested sample solution, and calibration curves were constructed. Analyses were duplicated to check the reproducibility of the results. Relative standard deviations among replicates were always less than 10%. Recovery tests were performed for the investigated metals in selected samples by spiking analyzed samples with aliquots of the metal standards and then carrying out digestion. The recovery percentages ranged from 96 to 99%. Throughout the manuscript, metal concentrations are presented as μg g^−1^ wet weight basis.

### 2.4. Health Risk Assessment

#### 2.4.1. Provisional Tolerable Weekly Intake (PTWI)

Dietary intake of Hg, Cd and Pb through seafood consumption was calculated using the following Equation (1):

EWI = (C × IR)/BW,(1)

where C represents the element concentration in seafood, IR the daily ingestion rate (g/day) of seafood (pelagic fish: 85 g week^–1^) [16] and body weight (70 kg). The estimated weekly intakes were compared with the Provisional Tolerable Weekly Intake (PTWI) of toxic elements (Hg: 4 µg kg^−1^ bw/week and 1.3 µg kg^−1^ bw/week for methylmercury (MeHg) [17]; Cd: 7 μg/kg bw/week and a tolerable weekly intake (TWI) of 2.5 μg/kg body weight established by EFSA [18]; Pb: 25 μg/kg bw/week [19]).

#### 2.4.2. Target Hazard Quotient (THQ)

The methodology for estimation of non-carcinogenic risk THQ and target carcinogenic risk was available in a U.S. EPA Region III Risk-based Concentration table [20,21] and it is described by the following Equation (2):

THQ = [(EF × ED × FIR × C/RfD × BW × AT)] × 10^−3^, (2)

where EF is exposure frequency (365 days/year); ED is exposure duration (80 years) [22], equivalent to the average lifetime; FIR is food ingestion rate (pelagic fish: 85 g week^–1^) [16]; C is metal concentration in fish (μg g^−1^); RfD is oral reference dose (Hg = 3.0 × 10^−4^ μg g^−1^ day^–1^, MeHg = 1.0 × 10^−4^ μg g^−1^ day^–1^, Cd = 1.0 × 10^−3^ μg g^−1^ day^–1^, Pb = 4.0 × 10^−3^ μg g^−1^ day^–1^, Zn = 3.0 × 10^−1^ μg g^−1^ day^–1^, Cu = 4.0 × 10^−2^ μg g^−1^ day^–1^, Ni = 2.0 × 10^−2^ μg g^−1^ day^–1^, Cr = 3.0 × 10^−3^ μg g^−1^ day^–1^) [20,21]; BW is body weight (70 kg); and AT is the averaging exposure time for non-carcinogens (365 days/year × ED). If the THQ value obtained is under “1,” an adverse effect is out of the question in terms of human health.

### 2.5. Statistical Analysis

The Kruskal–Wallis test was used to test the hypothesis about differences in the levels of metal accumulation, while a simple linear regression coefficient was used to examine the correlations between metals and specimen length. To investigate the influence of size on metal accumulation, the length of fish has been chosen because it is less subject to fluctuation than body weight [23]. The level of significance was set at *p* < 0.05.

## 3. Results and Discussion

### 3.1. Non-Essential Element Levels and Compliance with Permitted Legal Limits

**Figure 1:**
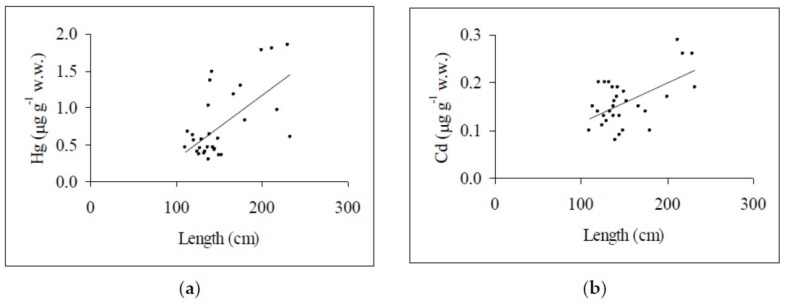
Correlation between Hg (**a**) and Cd (**b**) concentrations and length.

Concentrations (range, mean ± standard deviation and variance) of Hg, Cd, and Pb recorded in the swordfish specimens, as well as the respective permitted legal limits established by European Commission [24,25] for human consumption are illustrated in Table 1. The three toxic elements were detected in all samples examined, with Hg displaying the highest concentrations (mean: 0.77 μg g^−1^ wet weight), followed by Cd (mean: 0.16 μg g^−1^ wet weight) and Pb (mean: 0.11 μg g^−1^ wet weight) (*p* < 0.001). The analysis of variance showed a large intra-specific variability of Hg concentrations, while the opposite was verified for Cd and Pb. Chemical accumulation in marine organisms is influenced by an assortment of synergistic factors, including endogenous characteristics and physiological condition of the organism, feeding behavior, diet, geographical habitat, environmental features, and the metal’s tendency to undergo biomagnification in the food web. This latter property is typical of Hg, which enters the food chain via feeding organisms and gradually gets concentrated higher up the food chain [26], so an elevated load of Hg in these pelagic high trophic level predators is not surprising. The scientific literature confirms this assumption, also reporting that Hg concentrations are markedly higher than those found in our investigation. For example, surveys of swordfish specimens from different areas of the Mediterranean Sea, including the Ionian (1.58 μg g^−1^ wet weight) and Tyrrhenian Sea (1.04–2.41 μg g^−1^ wet weight), report elevated Hg concentrations [1], similar to other studies on swordfish from oceans around the world (Atlantic Ocean: 0.90–2.20 μg g^−1^ wet weight [27]), (Pacific Ocean: 1.81 μg g^−1^ wet weight [28]), (Indian Ocean: 1.30 μg g^−1^ wet weight [29]). However, caution is needed when comparing Hg concentrations because numerous factors influence the body burden of this metal. For example, variations in specimen size have a large effect on the magnitude of accumulated Hg, due to its propensity to increase with the size/length of the organisms. So the largest and potentially oldest fish exhibit higher Hg levels than younger organisms [27,30,31]. In complete accordance with this picture, results of linear regression analysis materialized a positive significant relationship between Hg concentrations and specimen length (*r* = 0.62; *p* < 0.001) (Figure 1a). Concerning Cd, there is evidence that this metal is less oriented towards accumulation in fish muscle tissue, where the concentrations are usually very low, preferring internal organs such as the liver and kidneys [32,33,34]. In the case in question, swordfish having a mixed diet consisting of fish but mainly of cephalopods [35], impregnated in Cd, showed relatively high concentrations consistent with those reported in specimens from different Mediterranean geographical locations (0.10–0.16 μg g^−1^ wet weight [1]) and the Indian (0.13 μg g^−1^ wet weight [36]) and Atlantic Ocean (0.14 μg g^−1^ wet weight [5]). Also for Pb, the contamination image was essentially similar to that encountered in swordfish specimens from Mediterranean waters around Corsica Island (0.08 μg g^−1^ wet weight [6]) and from the Ionian Sea along Italian coasts (0.05 μg g^−1^ wet weight [2]). For Pb, the concentrations did not vary with specimen size (*r* = 0.35; *p* > 0.05), while Cd levels revealed a length-dependent correlation (*r* = 0.56; *p* < 0.001) (Figure 1b). In general, accumulation of these two latter elements does not correspond with the age/size of marine organisms [14,37,38], however, the available literature data are rather controversial. Specimens from the Atlantic Ocean and Mediterranean Sea show a significantly positive correlation between body length and Pb and Cd concentrations [1], while swordfish from the Indian Ocean exhibit a positive correlation between Cd concentrations and length, but not for Pb [30]. These elements are extremely toxic even in trace amounts and, thus, the necessity of establishing hygienic standards for human consumption has been recognized by various countries in different opportunities. As depicted in Table 1, the European Community has established limits for Hg, which differ from one type of seafood to another, reaching a consumption limit of 1.0 μg g^−1^ wet weight for large predatory fish such as swordfish [39]. Also, for Pb and Cd, the limits have been updated and diversified according to different fishery products. For swordfish, values of 0.30 and 0.25 μg g^−1^ wet weight for Pb and Cd, respectively, have been fixed by the European Commission recently [24,25]. According to these standard limits, Hg, Cd, and Pb occurred at levels exceeding the respective critical values in eight, three, and two of the swordfish specimens examined, respectively. In samples non-compliant with the food safety regulations, the highest Cd and Pb concentrations found were 0.29 μg g^−1^ wet weight and 0.33 μg g^−1^ wet weight, respectively, which indicated that levels of these metals were exceedingly small. Conversely, Hg levels were higher than the regulatory limit, except in one sample in which the concentration of 1.02 μg g^−1^ wet weight was very close to the European safety standard. Concerning Hg, it is also important to emphasize that none of the swordfish smaller than 140 cm surpassed the maximum permitted by the European Community, except in one case.

### 3.2. Essential Element Levels and Compliance with Permitted Legal Limits

Concentrations (range, mean ± standard deviation, and variance) of Zn, Cu, Ni, and Cr recorded in the swordfish specimens as well as the respective permitted legal limits established in different countries for human consumption are illustrated in Table 1. For these elements the differences among concentrations reached levels of statistical significance (*p* < 0.001), with a distribution pattern following the sequence Zn > Cu > Ni > Cr. The data analysis also showed a large intra-specific variability of metal concentrations, with the strongest for Zn, whose values ranged from 3.38 to 15.74 μg g^−1^ wet weight (mean: 8.34 μg g^−1^ wet weight); intermediate for Cu, Ni, and Cr, whose levels varied from 0.30 to 1.87 μg g^−1^ wet weight (mean: 0.90 μg g^−1^ wet weight) and from 0.08 to 1.15 μg g^−1^ wet weight (mean: 0.52 μg g^−1^ wet weight), respectively, while Cr with concentrations between 0.03 and 0.22 μg g^−1^ wet weight (mean: 0.12 μg g^−1^ wet weight) showed the weakest variation. The concentrations of these essential metals in swordfish, on a global scale, are not well documented. However, the comparison reveals that our Cu and Cr values are similar to data encountered in Mediterranean specimens (Cu: 0.35 μg g^−1^ wet weight; Cr: 0.04 μg g^−1^ wet weight [6]) (Cu: 0.34 and 0.45 μg g^−1^ wet weight; Cr: 0.06 and 0.05 μg g^−1^ wet weight [40]), while, relative to Zn, Gobert et al. [6] report concentrations (30.28 μg g^−1^ wet weight) higher than those detected here. Remaining within the Mediterranean Sea, the present levels of Ni appear to be higher than those reported by Iamiceli et al. [40] (0.07 and 0.08 μg g^−1^ wet weight), but closer to the results illustrated by Gobert et al. [6] (0.27 μg g^−1^ wet weight). The values of Zn, Cu, and Cr in the fish investigated were also comparable to those reported by Bodin et al. [41] for swordfish from Seychelles waters (Indian Ocean), while for Ni the same author found much lower values (0.02 μg g^−1^ wet weight). With regard to the influence of size on essential metal levels, a linear regression analysis revealed that there was no accumulation pattern directly linked to length for Zn (*r* = 0.21; *p* = 0.28), Cu (*r* = 0.14; *p* = 0.46) and Cr (*r* = 0.14; *p* = 0.15), while a negative relationship was noted for Ni (*r* = 0.35; *p* > 0.05). Reports on this topic are scanty and fail to reach a general consensus as a consequence of the fact that the essential metals are subject to homeostatic regulation by species metabolism and consequently their accumulation is not correlated with the age/size of fish. However, Branco et al. [27] report a positive correlation between Zn concentrations and length in the blue shark, but not in swordfish. 

Kojadinovic et al. [30] observed an increase in Cu concentrations dependent on length in the muscle tissue of swordfish from the Indian Ocean, while the opposite finding was reported by Bodin et al. [41] in large pelagic fish species from the same geographical location. Similarly, Milatou et al. [42] found a negative relationships between Zn and Cu levels and Atlantic bluefin tuna specimen size. For these essential elements, legal thresholds are non-existent in Europe, while in different countries limits above which seafood is considered unsuitable for human consumption have been established. According to the U.K. Food Standards Committee’s Report, Zn and Cu should not exceed 50 and 20 μg g^−1^ wet weight [43], respectively. The Export Inspection Council of India states that in fish and fish products, the Ni level should not be more than 80 μg g^−1^ wet weight [44], while the Western Australian Food and Drug Regulation List sets a limit of 5.5 μg g^−1^ wet weight for Cr [45]. The results recorded here are compliant with these legal thresholds in all examined samples.

### 3.3. Assessment of Potential Public Health Risk

Recently, great interest has been paid to the investigation of toxic and essential trace element content in fish, as a result of growing concern about the health benefits and hazards associated with their consumption. In contrast to toxic metals, essential elements, in particular microelements, need to be consumed daily in adequate amounts for the maintenance of normal physiological processes in humans. However, deficiencies occur when they are consumed in insufficient quantities, and they may become toxic when taken in excessive amounts. So the changes in concentrations of essential elements as well as the presence of toxic metals, even in trace amounts, may cause various and strong metabolic alterations in humans. Consequently, it is crucial to monitor the levels not only of toxic metals, but also of essential elements in large species consumed, such as swordfish. As can be seen in Table 2, the calculated Hg, Cd, and Pb intakes, taking into account the mean contamination levels, constituted 23.3%, 2.7%, and 0.5% of the PTWIs, respectively, indicating that consumption of the species can be considered safe.

**Figure 2:**
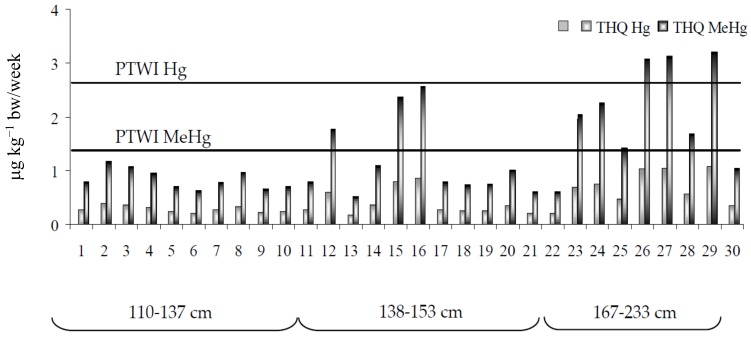
Provisional Tolerable Weekly Intake (PTWI) for Hg and MeHg and estimated weekly intake by consumption of different swordfish size spectra.

**Figure 3:**
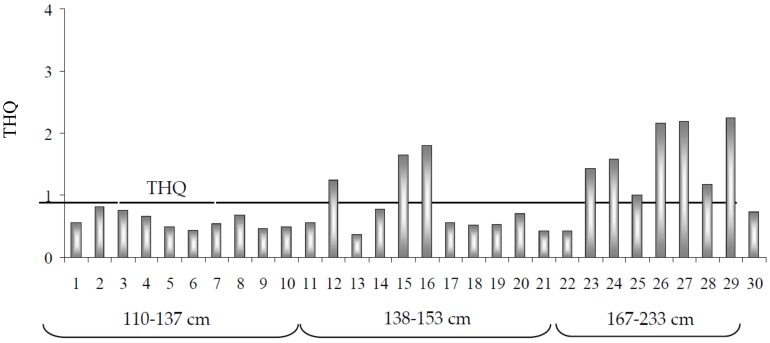
Estimated Target Hazard Quotient (THQ) for Hg and MeHg by consumption of different swordfish size spectra.

Also, the analysis of carcinogenic risk relative to all trace metals indicated a low health risk for consumers (THQ values < 1). However, because the assessment of exposure was estimated using mean metal concentrations, it is necessary to generate more accurate information, above all for Hg, whose concentrations change widely in relation to size. The consumption of specimens of smallest size (110–137 cm) determined intake values from 0.44 to 0.81 µg kg^−1^ bw/week, that of medium sized swordfish (138–153 cm) was associated with an intake between 0.36 and 1.80 µg kg^−1^ bw/week, while eating the largest fish (167–233 cm) led to an increase in exposure ranging from 0.73 to 2.25 µg kg^−1^ bw/week (Figure 2). These figures, representing a considerable percentage of PTWI (19.3–56.3%), need to be carefully evaluated in consideration of the fact that the present estimations did not include exposure from other foods. Furthermore, it should be pointed out that Hg accumulation is connected with that of MeHg, for which the European Food Safety Authority has set a Tolerable Weekly Intake of 1.3 µg kg^−1^ bw/week. According to this guideline and assuming that almost all Hg in muscle fish is present as MeHg, the scenario becomes alarming, because not only the consumption of larger fish, but also that of medium size specimens exceeds the safe weekly dose. Also, the estimated TEQ values for Hg and MeHg in relation to size categories indicate that consumers might have a higher probability of experiencing long-term hazardous effects (Figure 3). With respect to essential elements, the estimated daily intakes (EDI) remained lower than Dietary Reference Intakes (DRIs). In particular, Zn (1.3% for women; 0.9% for men) and Cu (1.1%) accounted for a small percentage of the DRI, while a major contribution was ascribed to Cr (5.8% for women; 4.2% for men), indicating that swordfish consumption constitutes a good source of this element.

## 4. Conclusions

The current study provides information on the concentrations of trace elements (Hg, Pb, Cd, Zn, Cu, Ni, and Cr) in swordfish, a commercially important species widely consumed due to its high-quality meat. The concentrations of essential elements were below the regulatory limits set by various extra-European countries, whereas toxic elements were under the legally defined limits in Europe, except for Hg, whose content varied widely from well below the maximum legal limit in smaller sized fish to levels substantially above the limit in larger fish. Such variation obviously reflects the exposure levels, which appear very high in relation to consumption of the largest fish. Also, the THQ values show that adverse human health effects might occur for ingestion of the largest fish. More research is needed to determine the fish size above which the Hg content exceeds the regulatory limit. This size threshold could be used to introduce size-specific catch limits to minimize health risk related to intake of this precious ichthyic resource.

## Figures and Tables

**Table 1:** Biometric data, metal concentrations (μg g^−1^ wet weight) (range, mean ± standard deviation, variance) and permitted legal limits.

Sample	Length (cm)	Weight (g)	Hg	Cd	Pb	Zn	Cu	Ni	Cr
1	110	14	0.46	0.10	0.09	15.74	0.75	1.15	0.22
2	114	16	0.67	0.15	0.12	6.44	1.33	0.44	0.11
3	120	18	0.62	0.14	0.05	9.12	0.47	0.66	0.11
4	121	17	0.55	0.20	0.13	8.43	1.50	0.99	0.15
5	125	21	0.40	0.11	0.11	5.75	0.38	0.80	0.04
6	127	20	0.36	0.13	0.09	9.30	0.84	0.40	0.06
7	128	23	0.45	0.20	0.08	8.65	0.37	0.33	0.17
8	130	30	0.56	0.12	0.10	7.07	0.61	0.28	0.06
9	133	33	0.38	0.20	0.11	8.04	0.43	0.78	0.09
10	134	35	0.40	0.14	0.09	3.38	0.40	0.79	0.17
11	137	42	0.46	0.19	0.11	8.92	1.31	0.32	0.08
12	137	40	1.02	0.13	0.33	8.22	1.78	0.93	0.16
13	138	43	0.30	0.15	0.09	9.39	1.11	0.46	0.07
14	139	42	0.63	0.16	0.07	8.61	1.41	0.12	0.15
15	140	44	1.36	0.08	0.08	6.48	0.67	0.08	0.19
16	142	43	1.48	0.17	0.03	7.25	1.87	0.68	0.03
17	143	44	0.46	0.19	0.02	8.70	0.30	1.01	0.08
18	145	45	0.42	0.09	0.10	8.35	1.09	0.49	0.07
19	145	44	0.43	0.13	0.16	15.15	0.41	0.55	0.13
20	149	46	0.58	0.10	0.07	9.53	0.50	0.73	0.11
21	150	49	0.35	0.18	0.06	7.45	0.95	0.17	0.17
22	153	53	0.35	0.16	0.13	6.35	0.34	0.17	0.04
23	167	66	1.18	0.15	0.09	7.05	0.75	0.88	0.10
24	175	83	1.30	0.14	0.05	8.60	1.10	0.10	0.14
25	180	92	0.82	0.10	0.09	13.96	0.77	0.69	0.10
26	200	108	1.78	0.17	0.09	7.88	1.50	0.09	0.13
27	212	113	1.80	0.29	0.12	7.23	1.15	0.28	0.21
28	218	120	0.97	0.26	0.15	6.59	0.98	0.98	0.15
29	230	127	1.85	0.26	0.16	5.56	0.44	0.12	0.13
30	233	130	0.60	0.19	0.31	7.00	1.41	0.08	0.19
Range	110–233	14–130	0.30–1.85	0.08–0.29	0.02–0.33	3.38–15.74	0.30–1.87	0.08–1.15	0.03–0.22
Mean ± St. Dev	153 ± 34	53 ± 35	0.77 ± 0.48	0.16 ± 0.05	0.11 ± 0.07	8.34 ± 2.62	0.90 ± 0.46	0.52 ± 0.33	0.12 ± 0.05
Variance	-	-	0.23	0.003	0.004	6.87	0.21	0.11	0.003
Permitted legal limits	-	-	1.0 ^1^	0.25 ^2^	0.30 ^3^	50.0 ^4^	20.0 ^4^	80.0 ^5^	5.5 ^6^

^1^ [39]; ^2^ [25]; ^3^ [24]; ^4^ [43]; ^5^ [44]; ^6^ Western Australian Food and Drug Regulation List [45].

**Table 2:** Estimation of the dietary intake for Hg, Cd, Pb, Zn, Cu, Ni, and Cr through swordfish consumption based on average concentrations.

	Hg	Cd	Pb	Zn	Cu	Ni	Cr
PTWI	4 ^1^	7 ^2^	25 ^3^	-	-	-	-
EWI	0.93	0.19	0.13	-	-	-	-
DRIs	-	-	-	11 (men); 8 (women)	0.9	-	35 ^4^ (men); 25 ^4^ (women)
EDI	-	-	-	0.10	0.01	-	1.46 ^4^
THQ	0.44	0.03	0.005	0.005	0.004	0.004	0.01

PTWI: Provisional Tolerable Weekly Intake (μg kg^−1^ body weight); EWI: Estimated Weekly Intake (μg kg^−1^ body weight); DRIs: Dietary Reference Intakes (mg day^−1^) [46]; EDI: Estimated Daily Intake; THQ: Target Hazard Quotient; ^1,2^ [17,18]; ^3^ [19]; ^4^ μg day^−1^.

## Supplementary Materials

The following are available online at http://www.mdpi.com/2305-6304/6/2/22/s1, Table S1: Lamp parameters for analysis of each element in terms of atomic absorption (Shimadzu AA 7000).

## References

[1] Damiano S., Papetti P., Menesatti P. (2011). Accumulation of heavy metals to assess the health status of swordfish in a comparative analysis of Mediterranean and Atlantic areas. Mar. Pollut. Bull..

[2] Renieri E.A., Alegakis A.K., Kiriakakis M., Vinceti M., Ozcagli E., Wilks M.F., Tsatsakis A.M. (2014). Cd, Pb and Hg Biomonitoring in Fish of the Mediterranean Region and Risk Estimations on Fish Consumption. Toxics.

[3] Storelli M.M., Giacominelli-Stuffler R., Storelli A., Marcotrigiano G.O. (2005). Accumulation of mercury, cadmium, lead and arsenic in swordfish and bluefin tuna from the Mediterranean Sea: A comparative study. Mar. Pollut. Bull..

[4] Lionetto M.G., Caricato R., Giordano M.E., Erroi E., Schettino T., Ekinci D. (2012). Carbonic Anhydrase and Heavy Metals. Biochemistry.

[5] Blanco S.L., González J.C., Vieites J.M. (2008). Mercury, cadmium and lead levels in samples of the main traded fish and shellfish species in Galicia, Spain. Food Add. Contam. Part B.

[6] Gobert S., Pasqualini V., Dijoux J., Lejeune P., Durieux E.D.H., Marengo M. (2017). Trace element concentrations in the apex predator swordfish (*Xiphias gladius*) from a Mediterranean fishery and risk assessment for consumers. Mar. Pollut. Bull..

[7] Olmedo P., Pla A., Hernández A.F., Barbier F., Ayouni L., Gil F. (2013). Determination of toxic elements (mercury, cadmium, lead, tin and arsenic) in fish and shellfish samples. Risk assessment for the consumers. Environ. Int..

[8] Rodrigues M.V., Yamatogi R.S., Sudano M.J., Galvao J.A., de Pérez A.C.A., Biondi G.F. (2013). Mercury Concentrations in South Atlantic Swordfish, *Xiphias gladius*, Caught off the Coast of Brazil. Bull. Environ. Contam. Toxicol..

[9] Storelli M.M., Barone G., Cuttone G., Giungato D., Garofalo R. (2010). Occurrence of Toxic Metals (Hg, Cd and Pb) in Fresh and Canned Tuna: Public Health Implications. Food Chem. Toxicol..

[10] European Food Safety Authority (EFSA) (2004). Opinion of the scientific panel on contaminants in food chain on a request from the commission related to mercury and methylmercury in food. EFSA J..

[11] European Food Safety Authority (EFSA) (2004). Press Release. EFSA Provides Risk Assessment on Mercury in Fish: Precautionary Advice Given to Vulnerable Groups. http://www.efsa.europa.eu/en/press/news/contam040318.htm/.

[12] Malak D.A., Livingstone S.R., Pollard D., Polidoro B.A., Cuttelod A., Bariche M., Bilecenoglu M., Carpenter K.E., Collette B.B., Francour P. (2011). Overview of the Conservation Status of the Marine Fishes of the Mediterranean Sea. The IUCN Red List of Threatened Species™–Regional Assessment. https://www.iucn.org/content/overview-conservation-status-marine-fishes-mediterranean-sea.

[13] Official Journal of the European Union (2017). Commission Regulation (EU) No. 644/2017 of 5 April 2017 Laying down Methods of Sampling and Analysis for the Control of Levels of Dioxins, Dioxin-Like PCBs and Non-Dioxin-Like PCBs in Certain Foodstuffs and Repealing Regulation (EU) No 589/2014. L 92/9. https://eur-lex.europa.eu/legal-content/EN/TXT/PDF/?uri=CELEX:32011R1259.

[14] Barone G., Giacominelli-Stuffler R., Storelli M.M. (2013). Comparative study on trace metal accumulation in the liver of two fish species (Torpedinidae): Concentration-size relationship. Ecotoxicol. Environ. Saf..

[15] Gazzetta Ufficiale Della Repubblica Italiana (GURI) (1994). Metodi di Analisi per la Ricerca di Residui di Metalli Pesanti e Arsenico.

[16] Food and Agriculture Organization (FAO) (2013). FAOSTAT Food Supply: Livestock and Fish Primary Equivalent.

[17] European Food Safety Authority (EFSA) (2012). Scientific Opinion on the risk for public health related to the presence of mercury and methylmercury in food. EFSA J..

[18] European Food Safety Authority (EFSA) (2009). Scientific opinion of the panel on contaminants in the food chain. Cadmium in food. EFSA J..

[19] Joint FAO/WHO Expert Committee on Food Additives (JECFA) (2000). Safety evaluation of certain food additives and contaminants. WHO Food Add. Ser..

[20] United States Environmental Protection Agency (US EPA) (2000). Risk Based Concentration Table.

[21] United States Environmental Protection Agency (US EPA) (2017). Risk Based Concentration Table.

[22] Demo Istat (2011). Speranza di Vita Alla Nascita e a 65 Anni, Per Sesso e Regione-Anni 2007–2010. http://demo.istat.it/altridati/indicatori/2010/Tab5.pdf.

[23] Diaz C., Galindo L., Garcia Montelongo F. (1994). Distribution of metals in some fishes from Santa Cruz de Tenerife, Canary Islands. Bull. Environ. Contam. Toxicol..

[24] Official Journal of the European Union (2015). Commission Regulation (EU) No. 1005/2015 of 25 June 2015 Amending Regulation (EC) No. 1881/2006 as Regards Maximum Levels of Lead in Certain Foodstuffs. L 161/9. http://eur-lex.europa.eu/legal-content/EN/TXT/PDF/?uri=CELEX:32015R1005.

[25] Official Journal of the European Union (2014). Commission Regulation (EU) No. 488/2014 of 12 May 2014 Amending Regulation (EC) No. 1881/2006 as Regards Maximum Levels of Cadmium in Foodstuffs. L 138/75. http://eur-lex.europa.eu/legal-content/EN/TXT/PDF/?uri=CELEX:32014R0488.

[26] Suhaimi F., Wong S.P., Lee V.L.L., Low L.K. (2006). Heavy metals in fish and shellfish found in local wet markets. Singapore J. Prim. Ind..

[27] Branco V., Vale C., Canário J., dos Santos M.N. (2007). Mercury and selenium in blue shark (*Prionace glauca*, L. 1758) and swordfish (*Xiphias gladius*, L. 1758) from two areas of the Atlantic Ocean. Environ. Pollut..

[28] Kumar M., Aalbersberg B., Mosley L. (2004). Mercury Levels in Fijian Seafood and Potential Health Implications.

[29] Chen M.H., Chen C.Y., Chang S.K., Huang S.W. (2007). Total and organic mercury concentrations in the white muscles of swordfish (*Xiphias gladius*) from the Indian and Atlantic oceans. Food Add. Contam..

[30] Kojadinovic J., Potier M., Le Corre M., Cosson R.P., Bustamante P. (2007). Bioaccumulation of trace elements in pelagic fish from the Western Indian Ocean. Environ. Pollut..

[31] Jinadasa B.K.K.K., Edirisinghe E.M.R.K.B., Wickramasinghe I. (2013). Total mercury content, weight and length relationship in swordfish (*Xiphias gladius*) in Sri Lanka. Food Add. Contam. Part B.

[32] Bonsignore M., Salvagio Manta D., Oliveri E., Sprovieri M., Basilone G., Bonanno A., Falco F., Traina A., Mazzola S. (2013). Mercury in fishes from Augusta Bay (Southern Italy): Risk assessment and health implication. Food Chem. Toxicol..

[33] Hussein A., Khaled A. (2014). Determination of metals in tuna species and bivalves from Alexandria, Egypt. Egypt. J. Aquat. Res..

[34] Storelli M.M., Barone G. (2013). Toxic Metals (Hg, Pb, and Cd) in Commercially Important Demersal Fish from Mediterranean Sea: Contamination Levels and Dietary Exposure Assessment. J. Food Sci..

[35] Stergiou K.I., Karpouzi V.S. (2002). Feeding habits and trophic levels of Mediterranean fish. Rev. Fish Biol. Fish..

[36] Jinadasa B.K.K.K., Rameesha L.R.S., Edirisinghe E.M.R.K.B., Rathnayake R.M.U.S.K. (2010). Mercury, Cadmium and Lead Levels in Three Commercially Important Marine Fish Species of in Sri Lanka. Sri Lanka J. Aquat. Sci..

[37] Canli M., Atli G. (2003). The relationships between heavy metal (Cd, Cr, Cu, Fe, Pb, Zn) levels and the size of six Mediterranean fish species. Environ. Pollut..

[38] Sakai H., Saeki K., Ichihashi H., Kamezaki N., Tanabe S., Tatsukawa R. (2000). Growth-Related Changes in Heavy Metal Accumulation in Green Turtle (*Chelonia mydas*) from Yaeyama Islands, Okinawa, Japan. Arch. Environ. Contam. Toxicol..

[39] Official Journal of the European Union (2008). Commission Regulation (EU) No. 629/2008 of 2 July 2008 Amending Regulation (EC) No. 1881/2006 Setting Maximum Levels for Certain Contaminants in Foodstuffs. L 173/6. http://eur-lex.europa.eu/legal-content/EN/TXT/PDF/?uri=CELEX:32008R0629.

[40] Iamaceli A.L., Ubaldi A., Lucchetti D., Brambilla G., Abate V., De Filippis E., Dellatte E., De Luca S., Ferri F., Fochi I. (2015). Metals in Mediterranean aquatic species. Mar. Pollut. Bull..

[41] Bodin N., Lesperance D., Albert R., Hollanda S., Michaud P., Degroote M., Churlaud C., Bustamante P. (2017). Trace elements in oceanic pelagic communities in the western Indian Ocean. Chemosphere.

[42] Milatou N., Dassenakis M., Megalofonou P. (2015). Do fattening process and biological parameters affect the accumulation of metals in Atlantic bluefin tuna?. Food Add. Contam. Part A.

[43] Ministry of Agriculture, Fisheries and Food (MAFF) (2000). Monitoring and Surveillance of Non-Radioactive Contaminants of Wastes at Sea, 1997.

[44] India Export Inspection Council (IEIC) (2002). Maximum Residual Limits (MRLs) for Pesticides, Heavy Metals and Antibiotics and Other Pharmacologically Active Substances in Fish and Fishery Products.

[45] Usero J., Izquierdo G., Morillo J., Gracia I. (2004). Heavy metals in fish (*Solea vulgaris*, *Anguilla anguilla* and *Liza aurata*) from salt marshes on the southern Atlantic coast of Spain. Environ. Int..

[46] Dietary Reference Intake (DRI) (2011). The Essential Guide to Nutrient Requirements Institute of Medicine (IOM).

