# Supplementary material for: Estimated Dietary Intake of Trace Metals from Swordfish Consumption: A Human Health Problem
Source: Toxics. 2018 Apr 3;6(2):22. doi: 10.3390/toxics6020022 (PMC6027446; doi:10.3390/toxics6020022)
Supplement: Supplementary file 1 [file toxics-06-00022-s001.pdf]

Supplementary Materials: Estimated Dietary Intake of Trace Metals from Swordfish Consumption: A Human Health Problem

Grazia Barone, Angela Dambrosio, Arianna Storelli, Rita Garofalo, Vito Pietro Busco and Maria Maddalena Storelli

Table S1. Lamp parameters for analysis of each element in terms of atomic absorption (Shimadzu AA 7000).

**Cd**	
BGC Mode	BGC-D2
Wavelength	228.8 nm
Lamp current	6 mA
Slit width	0.7 nm
**Cr**	
BGC Mode	BGC-D2
WaVelength	357.9 nm
Lamp current	10 mA
Slit width	0.7 nm
**Cu**	
BGC Mode	BGC-D2
Wavelength	324.8nm
Lamp current	8 mA
Slit width	0.7 nm
**Hg**	
BGC Mode	BGC-D2
Wavelength	253.7 nm
Lamp current	4 mA
Slit width	0.7 nm
No flame	HVG analysis
Burner heght	16 mm
**Ni**	
BGC Mode	BGC-D2
Wavelength	332.0 nm
Lamp current	12 mA
Slit width	0.2 nm
**Pb**	
BGC Mode	BGC-D2
WaVelength	283.3 nm
Lamp current	10 mA
Slit width	0.7 nm
**Zn**	
BGC Mode	BGC-D2
Wavelength	213.9 nm
Lamp current	8 mA
Slit width	0.7 nm
Flame type	Air–CH_2H_2
CH_2H_2 flow rate	2.0 L/min
Burner height	7 mm
